# Supplementary material for: Optimal dosing of dihydroartemisinin-piperaquine for seasonal malaria chemoprevention in young children
Source: Nat Commun. 2019 Jan 29;10:480. doi: 10.1038/s41467-019-08297-9 (PMC6351525; doi:10.1038/s41467-019-08297-9)
Supplement: Supplementary file 1 — Supplementary Information [file 41467_2019_8297_MOESM1_ESM.docx]

# Supplementary Information

# Optimal dosing of dihydroartemisinin-piperaquine for seasonal malaria chemoprevention in young children

Chotsiri, et al.

# Supplementary Figures


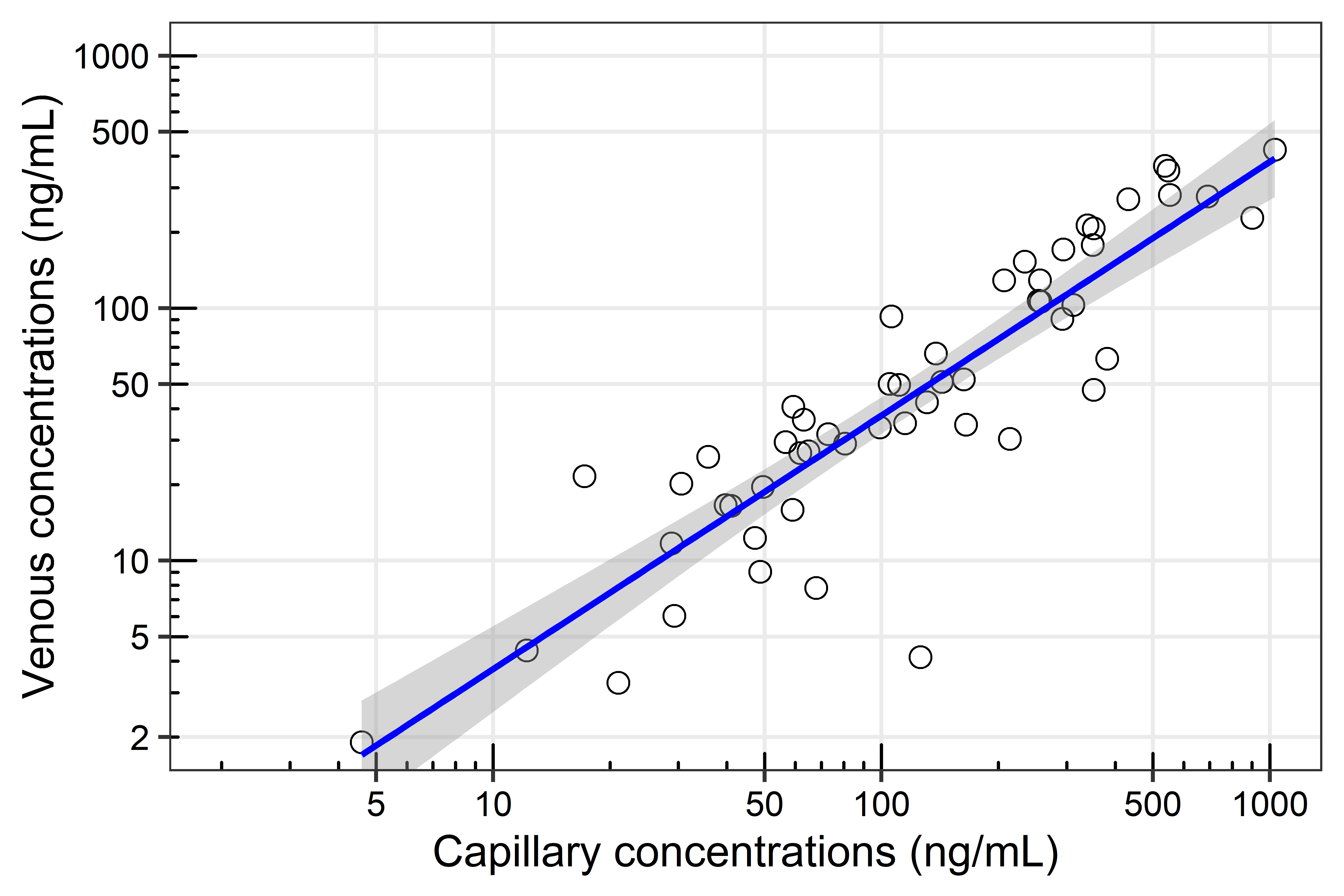


**Supplementary Figure 1. A linear regression of observed piperaquine capillary and venous plasma concentrations.** The solid blue line is a linear regression slope (predicted to be 0.4269; 95% CI: 0.3843 to 0.4695) and the shaded area is the 95% confidence interval of the predicted slope.


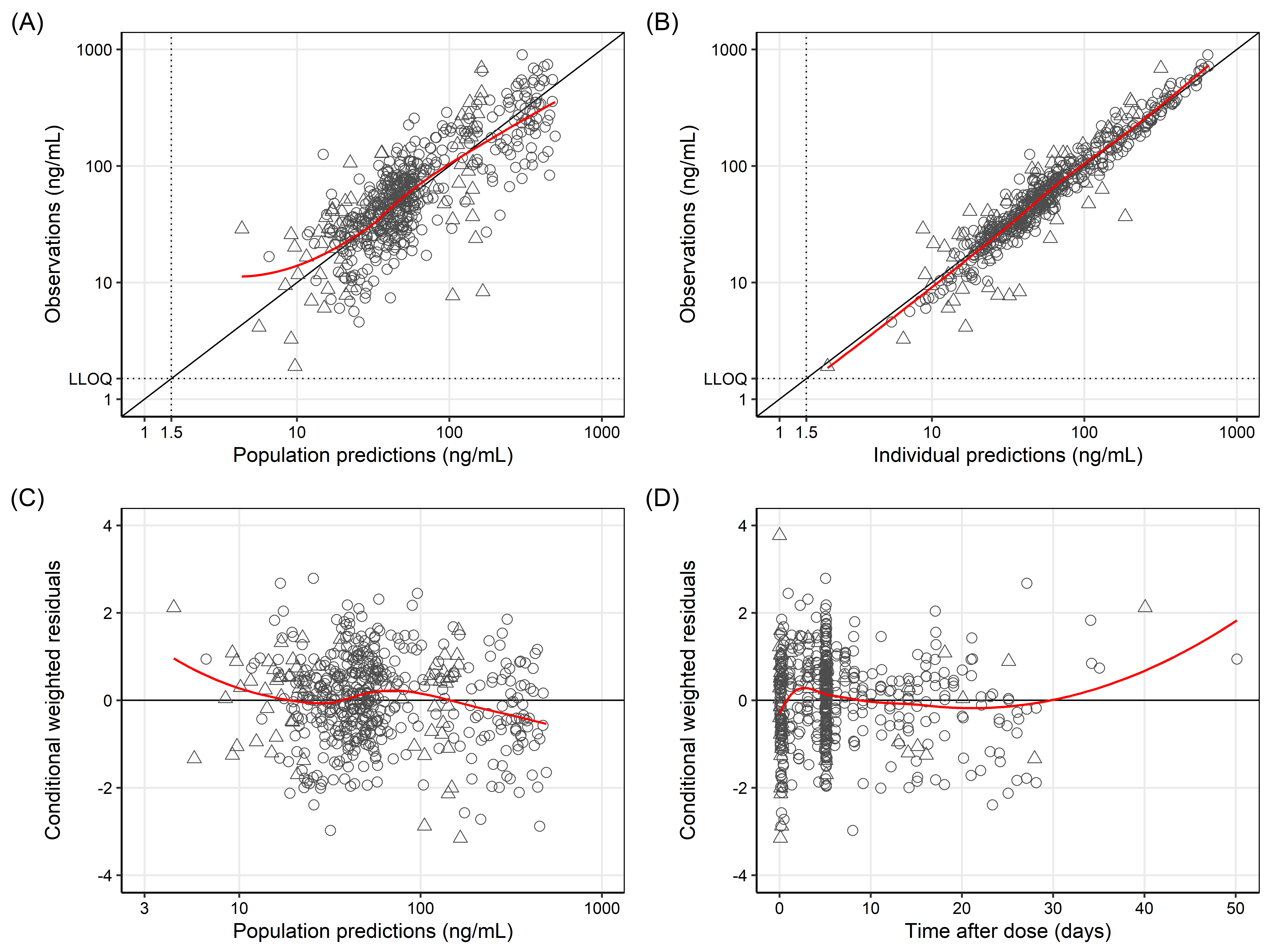


**Supplementary Figure 2. Goodness-of-fit plot of the final population pharmacokinetic model of piperaquine in children receiving dihydroartemisinin-piperaquine SMC.** (A) Observed piperaquine concentrations versus population predictions, (B) observed piperaquine concentrations versus individual predictions, (C) conditional weighted residuals versus population predictions, and (D) conditional weighted residuals versus time after dose. Open circles: observed capillary plasma concentrations; open triangles: observed venous plasma concentrations; solid lines: locally weighted least-squares regressions based on both capillary and venous data; horizontal dotted lines: lower limit of quantification (LLOQ) of piperaquine.

**Supplementary Figure 3.** **Relationship of parasite count at the time of detection (day 0) and the approximated start of the blood stage infection (i.e. time-to-event censoring interval).** The censoring interval begins (solid lines) by using a relatively low parasite density (~10^4^) and slow growth rate (~5-fold increase every 48 hours) back-extrapolation, and the censoring interval ends (dashed lines) by using a relatively high parasite density (~10^5^) and fast growth rate (~10-fold increase every 48 hours) back-extrapolation. This is illustrated by a patient (blue lines) presenting with a low parasite count (~10^8^) at detection, resulting in a start of the blood stage infection at 10.6-5.4 days before detection. Similarly a patient (red lines) with high parasite count (~10^13^) at detection, resulting in the start of the blood stage infection at 24.9-15.4 days before detection. The total parasite biomass was calculated as peripheral blood parasite count [μL^-1^] × body weight [kg] × 80,000.





**Supplementary Figure 4.** **Visual predictive plots of the interval-censoring time-to-event model of the internal data stratified by age groups.** (A) Age below 1.25 years, (B) age between 1.25 and 2.50 years, (C) age between 2.50 and 3.75 years, and (D) age above 3.75 years. The observed Kaplan-Meier plots are the black solid lines, and the shaded areas are the 95% prediction intervals of the final pharmacodynamic model.





**Supplementary Figure 5.** **Visual predictive plots of the interval-censoring time-to-event model of the external data stratified by age groups.** (A) Age below 1.25 years, (B) age between 1.25 and 2.50 years, (C) age between 2.50 and 3.75 years, and (D) age above 3.75 years. The observed Kaplan-Meier plots are the black solid lines, and the shaded areas are the 95% prediction intervals of the final pharmacodynamic model.


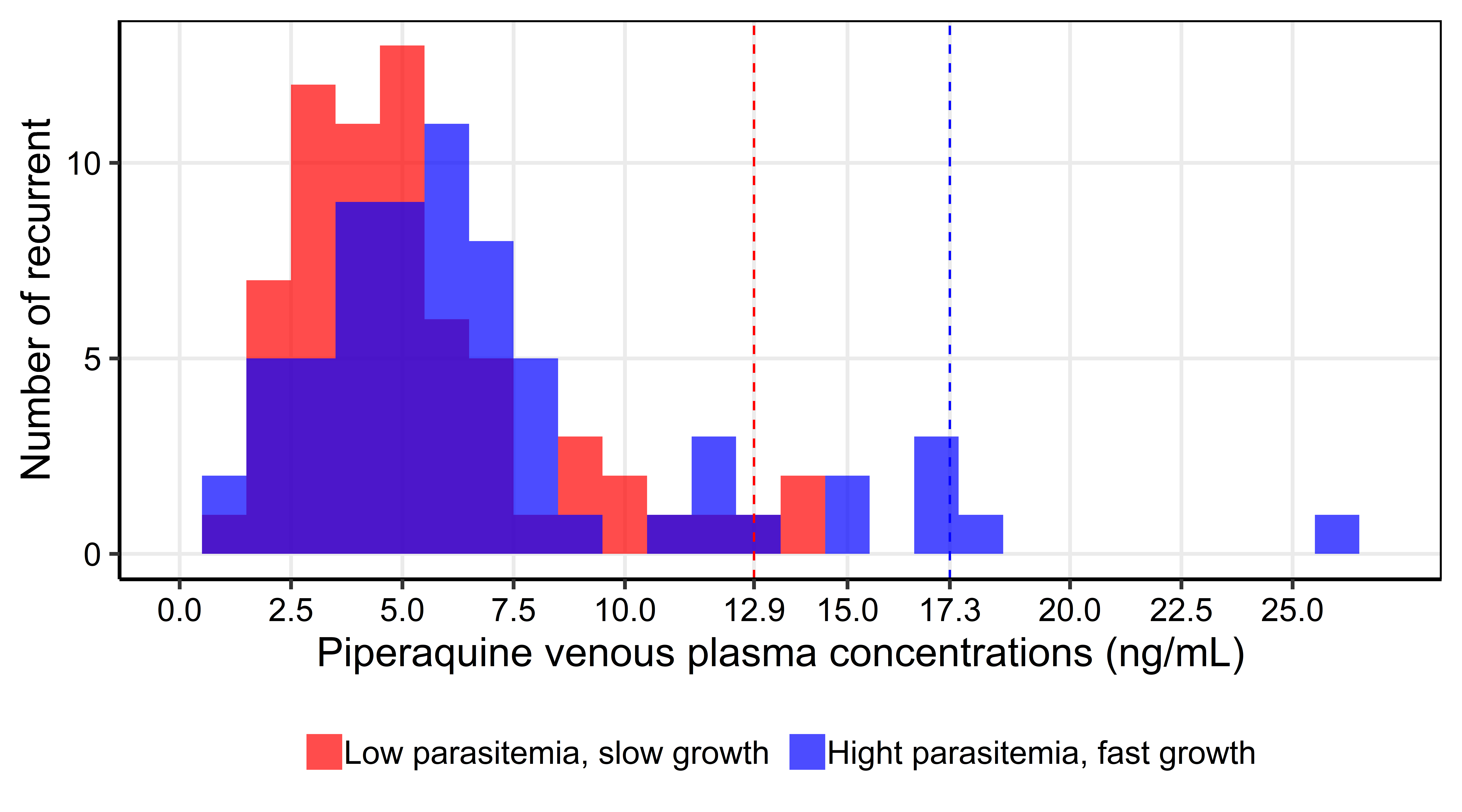


**Supplementary Figure 6.** **Histogram of the estimated minimum inhibitory concentration (MIC) of piperaquine in children with recurrent malaria.** Red bars represent the predicted venous piperaquine concentrations using a low blood stage parasite density (~10^4^) and slow growth rate (~5-fold increase every 48 hours) and blue bars represent the predicted venous piperaquine concentrations using a high blood stage parasite density (~10^5^) and fast growth rate (~10-fold increase every 48 hours). Vertical dashed lines represent the 95^th^ percentile of venous piperaquine concentrations under the two assumptions, assumed the lower and upper value of the *in vivo* minimum inhibitory concentration of piperaquine.

# Supplementary Table

**Supplementary Table 1.** **Secondary pharmacokinetic parameter estimates generated from the final pharmacokinetic-pharmacodynamic model of piperaquine in children receiving seasonal malaria chemoprevention in Burkina Faso.**

| Pharmacokinetic parameters | Median (95% CI) |
| --- | --- |
| C_max_ (ng/mL) | 398 (292-799) |
| T_max_ (h) | 2.85 (2.67-4.54) |
| t_1/2_ (days) | 21.3 (19.8-25.4) |
| AUC_0-30_ (h × ng/mL) | 31,200 (24,900-53,900) |
| AUC_30-60_ (h × ng/mL) | 38,300 (30,100-70,500) |
| AUC_60-90_ (h × ng/mL) | 40,600 (33,400-75,864) |
| Day-7 concentrations (ng/mL) after the first dose | 35.6 (29.3-68.3) |

Secondary parameter estimates are calculated from the empirical Bayesian *post hoc* estimates.

Abbreviations: AUC_0-30_, area under the capillary concentration-time curve from time 0 to 30 days; AUC_30-60_, area under the capillary concentration-time curve from time 30 to 60 days; AUC_60-90_, area under the capillary concentration-time curve from time 60 to 90 days; C_max_, maximum piperaquine capillary concentration; T_max_, time to maximum concentration; t_1/2_, terminal elimination half-life.

**Supplementary Table 2. Dosing regimen for translational simulations.**

| Body weight | Recommended dosing regimen (WHO 2010) | | | Increased dosing regimen (WHO 2015) | | |
| --- | --- | --- | --- | --- | --- | --- |
|  | **No. of tablets/day ^a^** | **PQP (mg)** | **PQP/day (mg/kg)** | **No. of tablets/day ^a^** | **PQP (mg)** | **PQP/day (mg/kg)** |
| 5 | 0.5 | 80 | 16.00 | 1 | 160 | 32.00 |
| 6 | 0.5 | 80 | 13.33 | 1 | 160 | 26.67 |
| 7 | 1 | 160 | 22.86 | 1 | 160 | 22.86 |
| 8 | 1 | 160 | 20.00 | 1.5 | 240 | 30.00 |
| 9 | 1 | 160 | 17.78 | 1.5 | 240 | 26.67 |
| 10 | 1 | 160 | 16.00 | 1.5 | 240 | 24.00 |
| 11 | 1 | 160 | 14.55 | 1.5 | 240 | 21.82 |
| 12 | 1 | 160 | 13.33 | 2 | 320 | 26.67 |
| 13 | 2 | 320 | 24.62 | 2 | 320 | 24.62 |
| 14 | 2 | 320 | 22.86 | 2 | 320 | 22.86 |
| 15 | 2 | 320 | 21.33 | 2 | 320 | 21.33 |
| 16 | 2 | 320 | 20.00 | 2 | 320 | 20.00 |
| 17 | 2 | 320 | 18.82 | 3 | 480 | 28.24 |
| 18 | 2 | 320 | 17.78 | 3 | 480 | 26.67 |
| 19 | 2 | 320 | 16.84 | 3 | 480 | 25.26 |
| 20 | 2 | 320 | 16.00 | 3 | 480 | 24.00 |

^a^ One tablet of the fixed dihydroartemisinin-piperaquine paediatric combination contained 20 mg dihydroartemisinin and 160 mg piperaquine tetra-phosphate (PQP) (equivalent to 85.72 mg piperaquine base).
