# Supplementary material for: Optimal dosing of dihydroartemisinin-piperaquine for seasonal malaria chemoprevention in young children
Source: Nat Commun. 2019 Jan 29;10:480. doi: 10.1038/s41467-019-08297-9 (PMC6351525; doi:10.1038/s41467-019-08297-9)
Supplement: Supplementary file 3 — Reporting Summary [file 41467_2019_8297_MOESM3_ESM.pdf]

nature research

Corresponding author(s): Joel Tarning

Last updated by author(s): Dec 7, 2018

# Reporting Summary

Nature Research wishes to improve the reproducibility of the work that we publish. This form provides structure for consistency and transparency in reporting. For further information on Nature Research policies, see Authors & Referees and the Editorial Policy Checklist.

## Statistics

For all statistical analyses, confirm that the following items are present in the figure legend, table legend, main text, or Methods section.

| n/a | Confirmed | |
|---|---|---|
| ☐ | ☒ | The exact sample size ($n$) for each experimental group/condition, given as a discrete number and unit of measurement |
| ☐ | ☒ | A statement on whether measurements were taken from distinct samples or whether the same sample was measured repeatedly |
| ☐ | ☒ | The statistical test(s) used AND whether they are one- or two-sided<br>*Only common tests should be described solely by name; describe more complex techniques in the Methods section.* |
| ☐ | ☒ | A description of all covariates tested |
| ☐ | ☒ | A description of any assumptions or corrections, such as tests of normality and adjustment for multiple comparisons |
| ☐ | ☒ | A full description of the statistical parameters including central tendency (e.g. means) or other basic estimates (e.g. regression coefficient) AND variation (e.g. standard deviation) or associated estimates of uncertainty (e.g. confidence intervals) |
| ☐ | ☒ | For null hypothesis testing, the test statistic (e.g. $F$, $t$, $r$) with confidence intervals, effect sizes, degrees of freedom and $P$ value noted<br>*Give P values as exact values whenever suitable.* |
| ☐ | ☒ | For Bayesian analysis, information on the choice of priors and Markov chain Monte Carlo settings |
| ☒ | ☐ | For hierarchical and complex designs, identification of the appropriate level for tests and full reporting of outcomes |
| ☒ | ☐ | Estimates of effect sizes (e.g. Cohen's $d$, Pearson's $r$), indicating how they were calculated |

*Our web collection on statistics for biologists contains articles on many of the points above.*

## Software and code

Policy information about availability of computer code

| | |
|---|---|
| Data collection | No software was used |
| Data analysis | Modelling and simulations were performed using NONMEM version 7.3. Piranha, and Perl-speaks-NONMEM (PsN version 4.4.0) was used for automation and diagnostics. R and ggplot package were used for visualising results. |

For manuscripts utilizing custom algorithms or software that are central to the research but not yet described in published literature, software must be made available to editors/reviewers. We strongly encourage code deposition in a community repository (e.g. GitHub). See the Nature Research guidelines for submitting code & software for further information.

## Data

Policy information about availability of data

All manuscripts must include a data availability statement. This statement should provide the following information, where applicable:

- Accession codes, unique identifiers, or web links for publicly available datasets
- A list of figures that have associated raw data
- A description of any restrictions on data availability

All relevant data and NONMEM code for the pharmacokinetic and pharmacodynamic model are available from the authors upon reasonable request.

# Field-specific reporting

Please select the one below that is the best fit for your research. If you are not sure, read the appropriate sections before making your selection.

☒ Life sciences ☐ Behavioural & social sciences ☐ Ecological, evolutionary & environmental sciences

# Life sciences study design

All studies must disclose on these points even when the disclosure is negative.

| | |
|---|---|
| Sample size | This is a descriptive study based on a subset data from previous clinical trial (Zongo I, et al., 2015). Sample size calculation is not applicable to this type of study. However, the sample size calculation was explained in the major clinical trial paper. |
| Data exclusions | The concentrations lower than the limit of quantification were excluded form the pharmacokinetic analysis. |
| Replication | The external model validation analysis in this study showed that the reproducibility of the finding are successful. |
| Randomization | The allocation of the drug regimen of the major clinical trial was random. However, this phamacokinetic-pharmacodynamic report was from one arm (dihydroartemisinin-piperaquine administered arm), the randomization is not applicable. |
| Blinding | Blinding is not relevant to this study. |

# Reporting for specific materials, systems and methods

We require information from authors about some types of materials, experimental systems and methods used in many studies. Here, indicate whether each material, system or method listed is relevant to your study. If you are not sure if a list item applies to your research, read the appropriate section before selecting a response.

## Materials & experimental systems

| n/a | Involved in the study |
|---|---|
| ☒ | ☐ Antibodies |
| ☒ | ☐ Eukaryotic cell lines |
| ☒ | ☐ Palaeontology |
| ☒ | ☐ Animals and other organisms |
| ☒ | ☐ Human research participants |
| ☐ | ☒ Clinical data |

## Methods

| n/a | Involved in the study |
|---|---|
| ☒ | ☐ ChIP-seq |
| ☒ | ☐ Flow cytometry |
| ☒ | ☐ MRI-based neuroimaging |

# Clinical data

Policy information about clinical studies

All manuscripts should comply with the ICMJE guidelines for publication of clinical research and a completed CONSORT checklist must be included with all submissions.

| | |
|---|---|
| Clinical trial registration | NCT00941785 |
| Study protocol | Study protocol was approved from the ethics committees of the London School of Hygiene and Tropical Medicine, United Kingdom and the ethics committee of the Centre Muraz (Comité d'Ethique Institutionnel du Centre Muraz), Burkina Faso. |
| Data collection | Data collection was performed at three rural health facilities in the district of Lena located 40-50 km from Bobo-Dioulasso, the second largest city of Burkina Faso. Children received a three-day fixed oral combination of DHA-PQ (Duocotexin, Beijing Holley-Cotec Pharmaceutical, China, 40 mg dihydroartemisinin and 320 mg piperaquine tetra-phosphate per ta blet) once a month for 3 consecutive months during the malaria seasonal (August – October, 2009). Blood samplings were allocated during the studied months (August – October, 2009). |
| Outcomes | The pharmacokinetic and pharmacodynamic model with parameter estimates are our expected outcomes. |
